# Action-Outcome Knowledge Dissociates From Behavior in Obsessive-Compulsive Disorder Following Contingency Degradation

**DOI:** 10.1016/j.bpsc.2018.09.014

**Published:** 2019-02

**Authors:** Matilde M. Vaghi, Rudolf N. Cardinal, Annemieke M. Apergis-Schoute, Naomi A. Fineberg, Akeem Sule, Trevor W. Robbins

**Affiliations:** a Department of Psychology, University of Cambridge, Cambridge, United Kingdom; b Behavioural and Clinical Neuroscience Institute, University of Cambridge, Cambridge, United Kingdom; c Department of Psychiatry, University of Cambridge, Cambridge, United Kingdom; d Liaison Psychiatry Service, Cambridgeshire and Peterborough National Health Service Foundation Trust, Cambridge, United Kingdom; e Hertfordshire Partnership University National Health Service Foundation Trust and University of Hertfordshire, Hertfordshire, United Kingdom; f Cumbria Partnership National Health Service Foundation Trust, National Health Service, Cumbria, United Kingdom

**Keywords:** Action-outcome, Frontostriatal, Goal-directed, Habit, Learning, Obsessive-compulsive disorder

## Abstract

**Background:**

In obsessive-compulsive disorder (OCD), actions persist despite being inappropriate to the situation and without relationship to the overall goal. Dysfunctional beliefs have traditionally been postulated to underlie this condition. More recently, OCD has been characterized in terms of an imbalance between the goal-directed and the habit systems. To test these competing hypotheses, we used a novel experimental task designed to test subjective action-outcome knowledge of the effectiveness of actions (i.e., instrumental contingency), together with the balance between goal-directed and habitual responding.

**Methods:**

Twenty-seven patients with OCD and 27 healthy control subjects were tested on a novel task involving the degradation of an action-outcome contingency. Sensitivity to instrumental contingency and the extent to which explicitly reported action-outcome knowledge guided behavior were probed by measuring response rate and subjectively reported judgments.

**Results:**

Patients with OCD responded more than healthy control subjects in situations in which an action was less causally related to obtaining an outcome. However, patients showed intact explicit action-outcome knowledge, as assessed by self-report. In patients, the relationship between causality judgment and responding was altered; therefore, their actions were dissociated from explicit action-outcome knowledge.

**Conclusions:**

These findings indicate reduced sensitivity to instrumental contingency in OCD, reinforcing the notion of a deficient goal-directed system in this disorder. By showing a dissociation between subjectively reported action-outcome knowledge and behavior, the data provide experimental evidence for the ego-dystonic nature of OCD.

Action is controlled by different learning mechanisms. Actions followed by a reinforcer are more likely to be repeated in the future in a habitual manner as a consequence of strengthening stimulus-response (S-R) representations. However, animals do not merely repeat previously reinforced actions but can instead make deliberate, goal-directed choices based on their knowledge of the relationship between an action and the associated outcome and their motivation to obtain that outcome (1). As such, adaptive everyday behavior is regulated by the balance between the goal-directed and the habitual systems. A disrupted balance between these two systems has been hypothesized to be relevant for understanding compulsive behaviors (2). These manifest as actions persistently repeated without relationship to the overall goal (3) where the habitual system seemingly overtakes response control (2).

Obsessive-compulsive disorder (OCD) is the prototypical disorder of compulsivity, and it manifests as a lack of goal-directed control over repetitive, ritualistic actions and intrusive thoughts. Compulsions are also characterized by the feeling of being compelled or forced to engage in such behaviors (4), and they are generally associated with the insight that such actions are ultimately harmful and purposeless. Therefore, OCD is ego-dystonic, as patients recognize their compulsive behaviors and thoughts as disproportionate, excessive, and maladaptive (5). Often, it is this disconnection between the responses patients find themselves making, as opposed to the responses they know to be rational, that causes so much distress (6).

Traditionally, cognitive theories posited dysfunctional beliefs as a major driver of OCD 7, 8. However, recent experimental evidence 9, 10, 11 has suggested that OCD is a disorder of habitual control, as irrelevant behavior is maintained in the face of changes in the value of the outcome previously associated with the action 9, 10, 11 and in the face of intact confidence updating (12). According to learning theory, goal-directed agents are also sensitive to the causal relationship (i.e., instrumental contingency) between the response and the reward: if instrumental responding continues when such contingencies are diminished, it is assumed to be under habitual (S-R) control (1). Contingency-based instrumental responding has been tested across species and found to be mediated by frontostriatal neural circuitry 13, 14, 15, 16, 17, 18, 19, 20, 21, 22 implicated in OCD (23) and other disorders of compulsivity [i.e., drug addiction (24) and binge-eating disorder (25)]. As the causal action-outcome association is weakened, a reduction in behavioral responding is usually observed, and, in humans, lower estimates of causal influence on outcome occurrence are reported via explicit causal judgments.

In this study, we capitalized on contingency degradation 1, 17 to test the robustness of action-outcome causal associations in OCD. With this experimental manipulation, we measured not only behavioral adjustment following changes in instrumental contingency but also how individuals subjectively perceived that causal relationship. Therefore, we were able to test whether patients with OCD, as compared with control subjects, 1) showed goal-directed control by modulating their behavior in response to changes in the causal action-outcome relationship; 2) accurately reported knowledge of the action-outcome causal relationship; and, crucially, 3) differentially used action-outcome knowledge to guide their behavior. Therefore, our experimental manipulation enabled testing the following two competing hypotheses of a correspondence versus a dissociation between perceived contingencies and behavior. On the one hand, compulsive behaviors (e.g., checking or rituals to prevent harm) may be interpreted as attempts to establish control. In this respect, compulsions might result from an increased sense of responsibility (7) or, in contrast, as superstitious behaviors carried out either to regain a subjective sense of control or because contingencies are misperceived 26, 27, 28. Accordingly, a correspondence between inflated (or deflated) perceived contingencies and behavior would argue for cognitive accounts for OCD, whereby compulsions are guided by erroneous cognitive interpretation of environmental cues. On the other hand, patients with OCD generally recognize their behavior as irrational and hence exhibit a dichotomy between their behavior and their beliefs about the effectiveness of their actions. Therefore, accurate action-outcome contingencies detection but imprecise behavioral adjustment to instrumental contingency would support a dissociation between an accurate cognitive appraisal of the environment and a failure to use this knowledge to guide behavior.

However, patients with OCD generally recognize their behavior as irrational and hence exhibit a dichotomy between their behavior and their beliefs about the effectiveness of their actions. Therefore, accurate action-outcome contingency detection but imprecise behavioral adjustment to instrumental contingency would support a dissociation between an accurate cognitive appraisal of the environment and a failure to use this knowledge to guide behavior. The ego-dystonic nature of OCD, whereby the urge to perform an action is associated with the knowledge that the action is excessive or irrelevant, would resonate with the latter scenario. In this study, we show this prediction to be valid.

## Methods and Materials

### Participants

The study included 27 patients and 27 healthy control subjects matched for relevant demographic variables (Table 1). Patients met criteria for OCD diagnosis with no current comorbidity. Patients were not enrolled if they scored less than 12 on the Yale-Brown Obsessive Compulsive Scale (29) and if they reported hoarding symptoms, as hoarding may represent a separate clinical entity (30). Nineteen patients were receiving medication for OCD (Supplement).

**Table 1:** Demographics and Clinical Characteristics of Studied Sample

	CTL Group (*n* = 27)	OCD Group (*n* = 27)	Statistic	*p*
Demographic Characteristics				
Gender, male/female	13/14	14/13	χ^2^_1_ = 0.074	.785
Age, years	40.67 ± 11.29	39.52 ± 10.65	*t*_52_ = 0.383	.704
Estimated verbal IQ^a^	117.99 ± 3.66	116.33 ± 3.45	*t*_51_ = 1.697	.096
Clinical Characteristics				
MADRS	1.11 ± 1.98	9.70 ± 4.65	*t*_52_ = −8.833	< .001
STAI-State^b^	28.15 ± 6.08	42.86 ± 9.97	*t*_51_ = −6.751	< .001
STAI-Trait^b^	33.85 ± 6.82	56.15 ± 7.96	*t*_51_ = −10.940	< .001
OCI-R^b^	6.37 ± 5.34	30.50 ± 12.33	*t*_51_ = −9.305	< .001
Y-BOCS total	—	22.52 ± 4.94		
Y-BOCS obsessions	—	10.81 ± 2.69		
Y-BOCS compulsions	—	11.70 ± 2.43		

Values are mean ± SD or *n*.

CTL, control; MADRS, Montgomery–Åsberg Depression Rating Scale; OCD, obsessive-compulsive disorder; OCI-R, Obsessive-Compulsive Inventory–Revised; STAI, State–Trait Anxiety Inventory; Y-BOCS, Yale-Brown Obsessive Compulsive Scale.

a Estimated verbal IQ was measured with the National Adult Reading Test; data from 1 participant with OCD were not included because English was not the participant’s mother tongue.

b Questionnaire data (OCI-R, STAI) from 1 participant with OCD were lost owing to technical error.

### Procedure

#### Contingency Degradation

**Figure 1:**
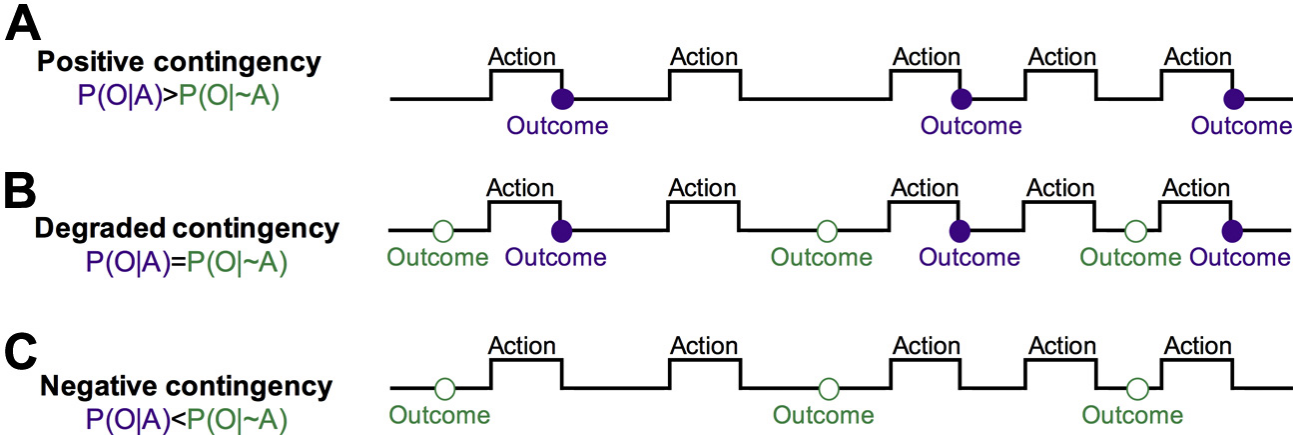
Contingency manipulation. To degrade the contingency, once agents have learned to perform an action to receive a reward with a certain probability, a schedule of noncontingent outcome delivery is superimposed. By increasing the frequency of noncontingent outcomes, the overall contingency (i.e., the causal association between an action and its consequences) is degraded or becomes negative. If guided by the goal-directed system, an agent should stop responding in the face of contingency degradation. **(A)** Diagram illustrating a schedule with a positive contingency, in which outcome is delivered on performance of an action with a given probability [P(O|A)]. **(B)** Contingency is degraded by also delivering outcomes in the absence of an action with a given probability [P(O|∼A)]. If the contingency is degraded to the extent that the two probabilities are equal, the causal status of the action is nil, and the probability of the reinforcer is the same regardless of any response. **(C)** When P(O|∼A) is higher than P(O|A), the contingency becomes negative, and the action reduces the probability of reinforcer delivery. P(O|A), probability of outcome given an action; P(O|∼A) probability of outcome given the absence of an action; violet filled circles, contingent outcomes; green empty circles, noncontingent outcomes.

We used contingency degradation experimental manipulation to study action-outcome contingencies detection. The standard contingency measure (1), ΔP, indexed the action-outcome relationship. ΔP was the difference between 1) the conditional probability of receiving an outcome following an action—that is, the probability of outcome given an action [P(O|A)], i.e., the probability of response-contingent outcome; and 2) the probability of receiving an outcome in the absence of that action—that is, the probability of outcome given the absence of an action [P(O|∼A)], i.e., the probability of a noncontingent outcome, such that ΔP = P(O|A) − P(O|∼A) (31). Measures of interest include the overall relationship between contingency and behavior and between contingency and perceived contingency. By increasing noncontingent outcomes, the contingency (i.e., the causal action-outcome association) is degraded, hence reduced, or becomes negative (Figure 1A–C). If guided by the goal-directed system, an agent should stop responding on contingency degradation.

#### Novel Protocol to Test Sensitivity to Action-Outcome Contingency

**Figure 2:**
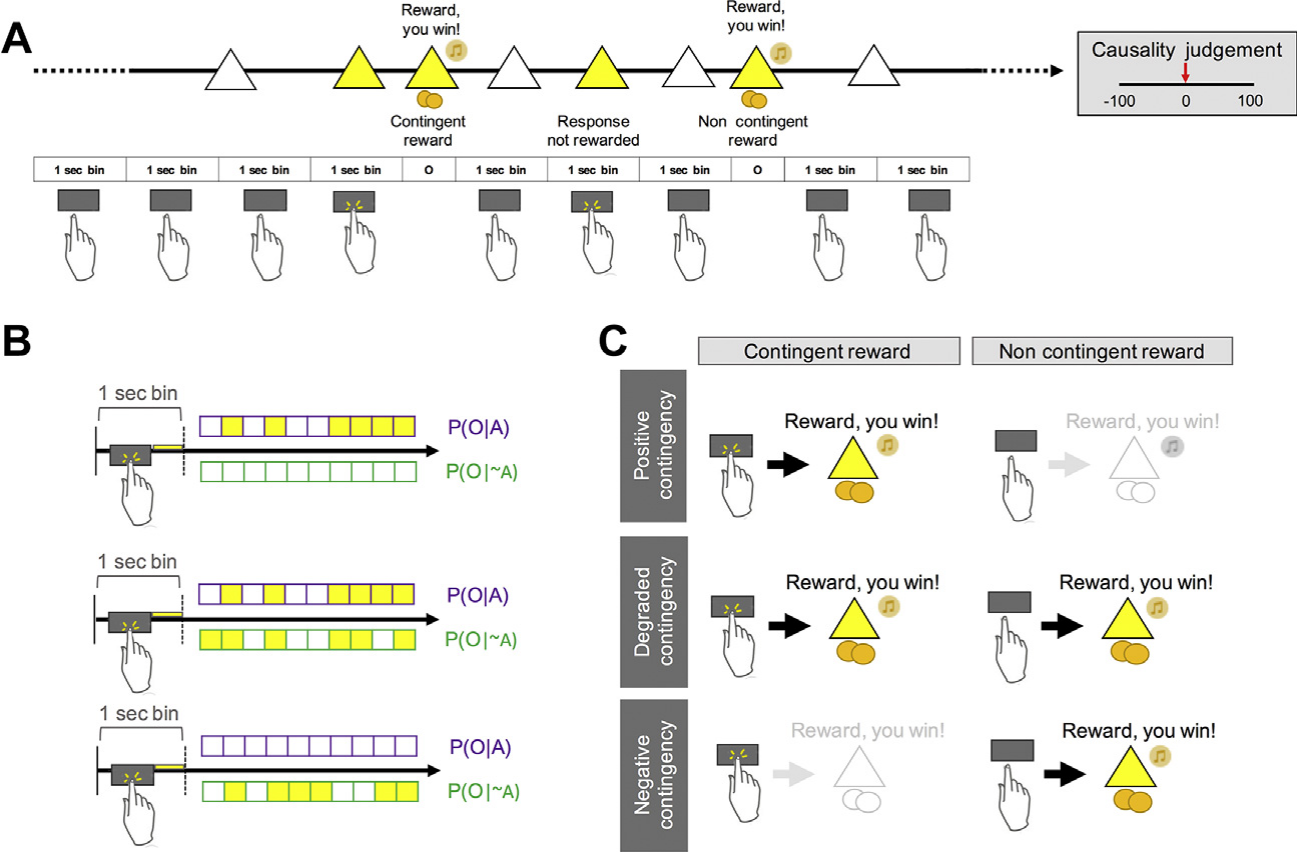
Experimental paradigm. **(A)** Subjects had to complete an experimental session of 12 blocks of 2 minutes each. At the end of each block, subjects had to judge to what extent pressing the space bar caused the occurrence of the reward, on a scale from −100 (pressing the space bar always prevented reward) to 100 (pressing the space bar always caused reward). During the experimental session, the participant was presented with a white triangle and could decide whether to press the space bar or not. Rewards were delivered contingently on pressing of the space bar or noncontingently in the absence of a response. In addition, a running total of the amount of money earned within a block was continuously displayed in the upper corner of the screen (not shown in figure). In cases where the participant was not pressing the space bar for multiple (hidden) 1-second bins in a row, the white triangle was continuously displayed on the screen, unless a nonresponse contingent reward occurred. In those cases, a reward was displayed on the screen noncontingently. **(B)** Each block was divided into 120 unsignaled time periods (bins) of 1 second. When a response occurred within each bin, the triangle turned yellow until the bin ended. If a response was recorded during the bin, a contingent reward was delivered at the end of that bin according to the applicable probability of outcome delivery given a response [P(O|A)]. If no response occurred during the bin, a noncontingent reward was delivered according to the applicable probability of outcome delivery given the absence of a response [P(O|∼A)]. **(C)** By varying P(O|A) and P(O|∼A), different levels of contingencies were achieved so that each experimental session included positive, degraded, and negative contingency blocks. O, outcome; P(O|A), probability of outcome given an action; P(O|∼A), probability of outcome given the absence of an action.

We developed a novel free-operant, self-paced procedure (Supplement). A white triangle permanently on the screen signaled that the participant was free to press or not press the space bar. When a reward was delivered, following a key press or not, a yellow triangle with a 25p image was shown at the end of the bin for 500 ms with the text “Reward, you win!” and a tone (Figure 2A). On each response, the triangle turned yellow until the end of the a priori specified bin to signal that a response has been recorded and to prevent multiple responses within the same 1-second bin. If no outcome was delivered, no feedback was given, and the next bin started. A running total of pence accumulated within the block was displayed in the top right corner of the screen. There were 12 blocks, not explicitly labeled as such to the participants. At the beginning of each block, the running total of pence was reset to 0. Causality judgments on the relationship between pressing the key and receiving the reward were collected at the end of each block (Figure 2A). The first 3 blocks (blocks 1–3) were always presented in the same order (high contingency, degradation, extinction), providing an implicit training phase. The remaining blocks (i.e., blocks 4–12) were presented according to a Latin square design across participants (Table 2). Each block lasted for 2 minutes (120 unsignaled bins) (Supplement). If a response occurred during a given bin, the outcome was delivered with probability P(O|A) defined a priori for that block; if no response occurred, the outcome was delivered with probability P(O|∼A) defined a priori for that block (Figure 2B). Only the first space-bar press within the bin had any programmed consequences. The total number of responses within each bin was also recorded, but responding beyond the first response of the bin had no programmed consequences. We varied P(O|A) and P(O|∼A) giving blocks with different contingency levels and obtaining different experimental conditions (Figure 2B, C and Table 2). We did not include a cost for responding, in line with experimental studies in rodents where there is no explicit punishment for responding (see Supplementary Pilot Experiments and Supplemental Figure S3 for results from pilot experiments with and without such costs).

**Table 2:** Response Rates and Causality Judgments

Block		Programmed Contingency			Experienced Contingency		Response Rate		Causality Judgment	
		P(O|A)	P(O|∼A)	ΔP	CTL	OCD	CTL	OCD	CTL	OCD
Fixed Order	1	0.60	0.00	0.60	0.59	0.60	0.51 (0.21)	0.49 (0.24)	43.30 (34.27)	48.60 (31.82)
	2	0.60	0.60	0.00	0.01	0.05	0.26 (0.20)	0.35 (0.27)	8.17 (27.64)	10.67 (44.74)
	3	0.00	0.00	0.00	0.00	0.00	0.37 (0.27)	0.48 (0.27)	−10.46 (39.75)	−14.81 (45.60)
Shuffled in Latin Square Design	4	0.00	0.00	0.00	0.00	0.00	0.38 (0.23)	0.48 (0.27)	−15.17 (36.06)	−21.35 (40.43)
	5	0.00	0.30	−0.30	−0.29	−0.30	0.26 (0.21)	0.27 (0.21)	−50.43 (47.60)	−41.51 (55.30)
	6	0.00	0.60	−0.60	−0.62	−0.60	0.20 (0.23)	0.21 (0.16)	−55.27 (44.83)	−32.53 (66.08)
	7	0.30	0.00	0.30	0.30	0.30	0.49 (0.22)	0.62 (0.20)	27.54 (24.20)	35.34 (25.33)
	8	0.30	0.30	0.00	0.03	0.00	0.34 (0.26)	0.41 (0.24)	0.01 (31.53)	0.36 (37.34)
	9	0.30	0.60	−0.30	−0.28	−0.29	0.29 (0.26)	0.32 (0.26)	−12.95 (47.98)	−9.40 (38.56)
	10	0.60	0.00	0.60	0.60	0.60	0.62 (0.21)	0.56 (0.23)	56.01 (26.67)	53.52 (30.84)
	11	0.60	0.30	0.30	0.32	0.31	0.38 (0.26)	0.53 (0.25)	22.49 (30.71)	33.06 (32.79)
	12	0.60	0.60	0.00	−0.01	0.00	0.29 (0.23)	0.38 (0.25)	9.64 (31.81)	8.30 (36.75)

Dependent variables are given as mean (SD). Blocks 1–3 were presented in a fixed order; blocks 4–12 were presented according to a Latin square design. Lines in the table refer to different conditions depending on the density of contingent outcomes. Block 4, block 5, and block 6 had P(O|A) = 0.0; block 7, block 8, and block 9 had P(O|A) = 0.3; block 10, block 11, and block 12 had P(O|A) = 0.6. Programmed contingency refers to the a priori experimentally programmed contingency resulting from the a priori programmed conditional probabilities.

CTL, control; OCD, obsessive-compulsive disorder; P(O|A), probability of outcome given an action; P(O|∼A), probability of outcome given the absence of an action; ΔP, contingency.

#### Experienced Contingency

As expected based on our task’s implementation, there was a high correlation between the mean experienced contingency (based on experienced event frequencies) and the contingencies programmed a priori for control subjects (*r* = .999, *p* < .001) and patients (*r* = .998, *p* < .001) alike (Table 2 and Supplement). Therefore, programmed contingencies were used for subsequent analysis. Our findings were not confounded by between-group differences in experienced contingencies, as no main effect of group (*F*_1,48.49_ = 0.01, *p* = .940) or interaction between group and block (*F*_11,559.95_ = 1.06, *p* = .395) on experienced contingency was found.

### Data Analysis

All statistical tests were two-sided, and parametric or nonparametric tests were applied according to assumptions of the specific statistical test. We analyzed response rate and causality judgments using a two-step approach. First, we identified if there was a between-group difference in sensitivity to instrumental contingency. To this end, we computed a response rate by dividing the number of responses by the number of bins for each block. For each dependent variable (response rate and causality judgment), programmed contingency was used as a within-subject factor, and group was used as a between-subject factor. All data were collapsed across blocks having equal contingencies. Analyses were performed in R version 3.3.1 (R Foundation for Statistical Computing, Vienna, Austria; http://www.r-project.org/) using the ez package for analysis of variance. Levene’s test was used to verify homogeneity of variance. Mauchly’s test of sphericity was applied, and Greenhouse-Geisser and Huynh-Feldt corrections were used for substantial (ε < 0.75) and minimal (ε ≥ 0.75) violation, respectively. To investigate the relationship between contingency judgments and response rate between groups, we used linear mixed-effects models. Group was used as a fixed-effect factor; linear (and, where applicable, quadratic) causality judgments were used as continuous fixed-effect predictors. The maximal random-effect structure justified by the design was specified (32) using mixed models (33).

Second, for conditions in which we observed diminished sensitivity to instrumental contingency in patients, we tested whether such behavior was due to imbalances in the goal-directed and habit systems. Accordingly, we obtained a ratio score by considering pairs of contingent and corresponding degraded blocks in which P(O|A) was stable and P(O|∼A) was increased (18). Therefore, we also focused on specific contingency transitions in which P(O|∼A) increases without changes to P(O|A): this manipulation degrades instrumental contingency without affecting the contiguity of actions and outcomes that drives S-R habits, so it is a specific test for excessive habitual responding. For each pair, the number of responses in the contingent block was divided by the sum of responses in both the contingent and the degraded blocks. Thus, the ratio score represents the number of responses in the contingent condition as a proportion of the total responses made across both contingent and degraded conditions. Values close to 1 indicate high sensitivity to contingency, and values close to 0.5 indicate habitual behavior indexing equal responding in both contingent and correspondingly degraded conditions.

## Results

### Effect of Instrumental Contingency on Response Rate

**Figure 3:**
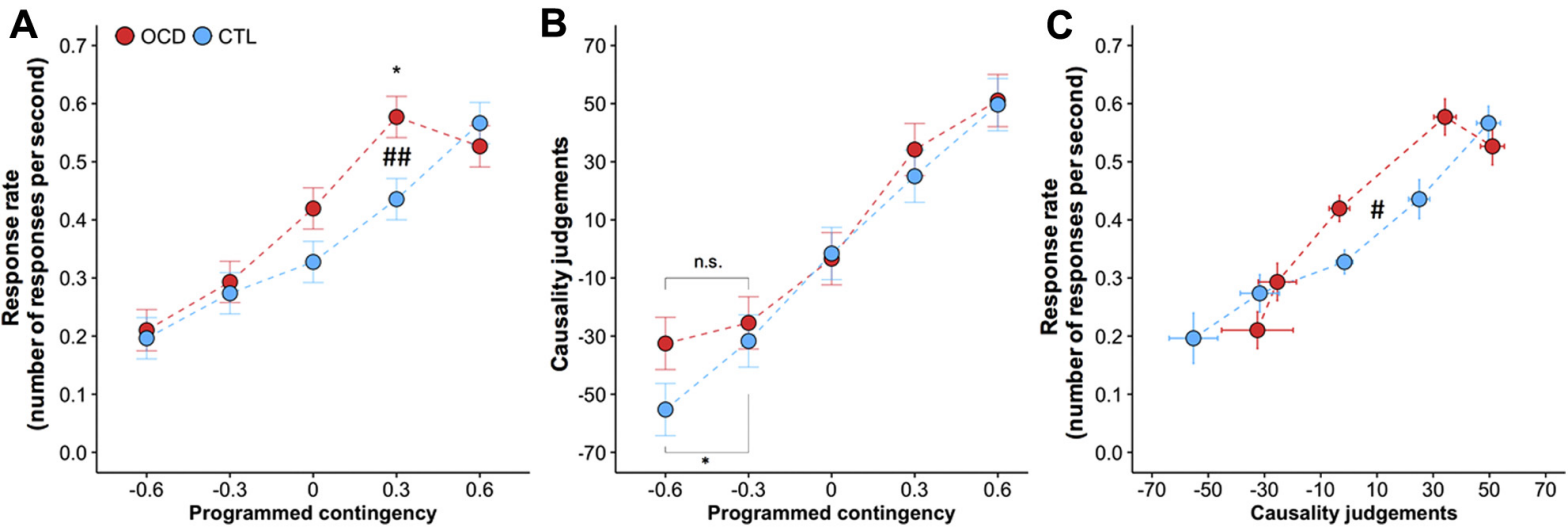
Mean response rate and causality judgments for control (CTL) group and obsessive-compulsive disorder (OCD) group. **(A)** Mean response rate by contingency (ΔP). Both groups responded more for higher contingencies. However, patients with OCD showed reduced sensitivity to instrumental contingency. **(B)** Subjective judgments of causality increased as a direct function of response-outcome contingency in both groups. Data are presented in ascending order of programmed contingency, but contingencies were experienced by each subject in a semirandomized order. Error bar indicates Fisher’s least significant difference to facilitate post hoc comparisons (error bars are ± 0.5 least significant difference). However, in the context of mixed designs, as in this case, this error bar can be used only for within-subject comparisons. The difference between OCD and CTL groups in mean causality judgments at ΔP = −0.6 was not significant. However, CTL subjects, but not patients with OCD, subjectively detected a difference between neighboring levels of negative programmed contingency between ΔP = −0.3 and ΔP = −0.6). **(C)** Response rate as a function of causality judgment by group. The two populations differentially employed action-outcome knowledge to guide their behavior. Points and error bars (SEMs) show values clustered by programmed contingency. As described in the main text, data were collapsed across blocks having equal contingencies (ΔP = −0.6, block 6; ΔP = −0.3, block 5, block 9; ΔP = 0.0, block 2, block 3, block 4, block 8, block 12; ΔP = 0.3, block 7, block 11; ΔP = 0.6, block 1, block 10. See Table 2 for naming of the blocks). Programmed contingency refers to the a priori experimentally programmed contingency resulting from the a priori programmed conditional probabilities. *p < .05, within-group comparison; ^##^*p* < .01, interaction; ^#^*p* < .05, group × quadratic causality judgment interaction. n.s., not significant.

Mean response rate increased with contingency (*F*_4,208_ = 65.028, *p* < .001) (Figure 3A). Overall, there were no between-group differences in response rate (*F*_1,52_ = 1.074, *p* = .305), ruling out apathy or generalized impulsivity in patients. Between-group responding was differentially affected by the contingency (group × contingency: *F*_4,208_ = 3.922, *p* = .01); this difference was explored via between-group simple-effect comparisons at each contingency level. Patients persisted in responding more than control subjects with low instrumental contingency (ΔP = 0.3, *F*_1,52_ = 6.036, *p* = .017). Such responding did not correlate with impulsivity, as measured by the Barratt Impulsiveness Scale (*r* = .312, *p* = .129) (34). Patients also responded marginally more at ΔP = 0.0, but this did not reach significance (*F*_1,52_ = 3.185, *p* = .080). Results were not affected by medication status or by reward responsiveness (Supplement and Supplemental Figure S1).

Additional responding within each 1-second time bin (superfluous responses beyond the first, i.e., with no behavioral effect) was not affected by instrumental contingency (contingency: *F*_4,208_ = 0.621, *p* = .648) or group (group: *F*_1,52_ = 0.017, *p* = .896; group × contingency: *F*_4,208_ = 0.070, *p* = .991). Differences in the additional number of responses within each bin would have been consistent with a framework in which excessive responding in OCD is attributed to inhibition failure. Our findings instead reinforced the notion that patients expressed habitual responding, a hypothesis we test directly below.

### Effect of Instrumental Contingency on Causality Judgments

Causality ratings were a direct function of action-outcome contingency (*F*_4,208_ = 74.099, *p* < .001) (Figure 3B). There were no between-group differences in causality judgments (group: *F*_1,52_ = 2.379, *p* = .129; group × contingency: *F*_4,208_ = 1.084, *p* = .366). Medication status did not affect the results (Supplement and Supplemental Figure S1).

### Relationship Between Response Rate and Causality Judgments

Patients and control subjects differed in the way causality judgments predicted response rate, in a nonlinear fashion. Overall, response rate was linearly predicted by causality ratings (*F*_1,45.449_ = 58.154, *p* < .001), with no between-group difference (group × causality_linear_: *F*_1,45.449_ = 1.489, *p* = .229). However, there was a significant nonlinear effect as well, which differed between groups (group × causality_quadratic_: *F*_1,204.827_ = 3.959, *p* = .0479) (Figure 3C). Although model fitting residuals indicated larger residual variance in the OCD group (*F*_323,323_ = 1.28, *p* = .013), this has no obvious implications for altering the interpretation (35).

This analysis thus indicated an altered, nonlinear relationship between causality judgments and response rate in patients and represents a formal demonstration of the differential use of action-outcome knowledge to modulate behavior in patients, also supported by patients’ reports (Table 3). Thus, in patients, for positive contingencies, behavior persisted for low instrumental contingencies despite intact and accurately reported action-outcome knowledge of the causal effect of their actions. For negative contingencies, the equivalent response rates between control subjects and patients (Figure 3A) interacted with a tendency for patients to believe their actions to be less detrimental than control subjects (programmed ΔP = −0.3 and ΔP = −0.6) (Figure 3B). Therefore, patients did not recognize their action to have very negative consequences. However, they behaved the same way as control subjects, whose ratings correctly identified a highly negative action-outcome contingency. We analyzed response rate for different time windows of each block, thus excluding the possibility that such dissociation was due to different learning processes in patients (Supplement and Supplemental Figure S2).

**Table 3:** Subjective Accounts When the Contingency Was Zero

	Subjective Accounts of Behavior Adopted (Multiple Choice)									
	Other		Mostly Did Not Press		Sometimes Pressed		Kept Pressing			
CTL^a^	2		17		7		1		χ^2^ = 17.839, *p* < .001	
OCD^a^	4		3		5		10			

	Subjective Accounts of Behavior Adopted (Summary of Spontaneous Descriptions)									
	CTL	OCD^b^	CTL	OCD^c^	CTL	OCD^d^	CTL	OCD^e^	CTL^f^	OCD^f^
No point/no difference (Pressing or not did not make any difference)	1	—	14	2	2	3	—	1	63%	27%
Checking (To check whether occurrence of reward changed)	1	2	1	—	2	—	—	1	15%	14%
Habit (Can’t stop/In the habit of pressing)	—	—	—	—	—	—	—	2	0%	9%
Just in case (Just in case reward stopped when not pressing the bar)	—	—	—	1	—	1	—	1	0%	14%
Mind wandering (Kept pressing because mind wandering)	—	—	—	—	—	—	—	1	0%	4%
Other^g^	—	2	2	—	3	1	1	4	22%	32%

CTL, control; OCD, obsessive-compulsive disorder.

a Absence of contingency identified CTL participants (27/27) and OCD participants (22/22). Data were not available for 5 participants with OCD. Control participants and participants with OCD recognized the absence of contingency in relevant blocks and that key pressing did not make a difference. The majority of control participants did not press the key. In contrast, more participants with OCD continued to press the key. Subjective accounts for behavior adopted also differed, with the majority of control participants giving as a reason that pressing or not pressing made no difference to the occurrence of the outcome. In contrast, a minority of participants with OCD gave this subjective account; the majority justified their behavior instead as checking, habit, or “just in case” conduct.

b--e Each pair refers to the spontaneous description of the corresponding multiple choice category (^*b*^Other; ^*c*^Mostly Did not Press; ^*d*^Sometimes Pressed; ^*e*^Kept Pressing).

f Percentage corresponds to the proportion of people within each group to give a specific subjective account of behavior. Percentage was calculated based on the number of people within each group correctly identifying an absence of contingency (CTL, *n* = 27; OCD, *n* = 22).

g Seven participants with OCD gave subjective accounts that were classified as “Other” (2, “Don’t know”; 1, “I pressed the space bar because it was less boring”; 1, “Pressing was entertaining and did not cause any loss”; 1, “I pressed sometimes according to the feeling of what it was better”; 1, “I pressed because the money was occasionally coming”; 1, “I pressed the space bar sometimes pressed because otherwise nothing was happening”).

### Habit or Goal-Directed Ratio Score

**Figure 4:**
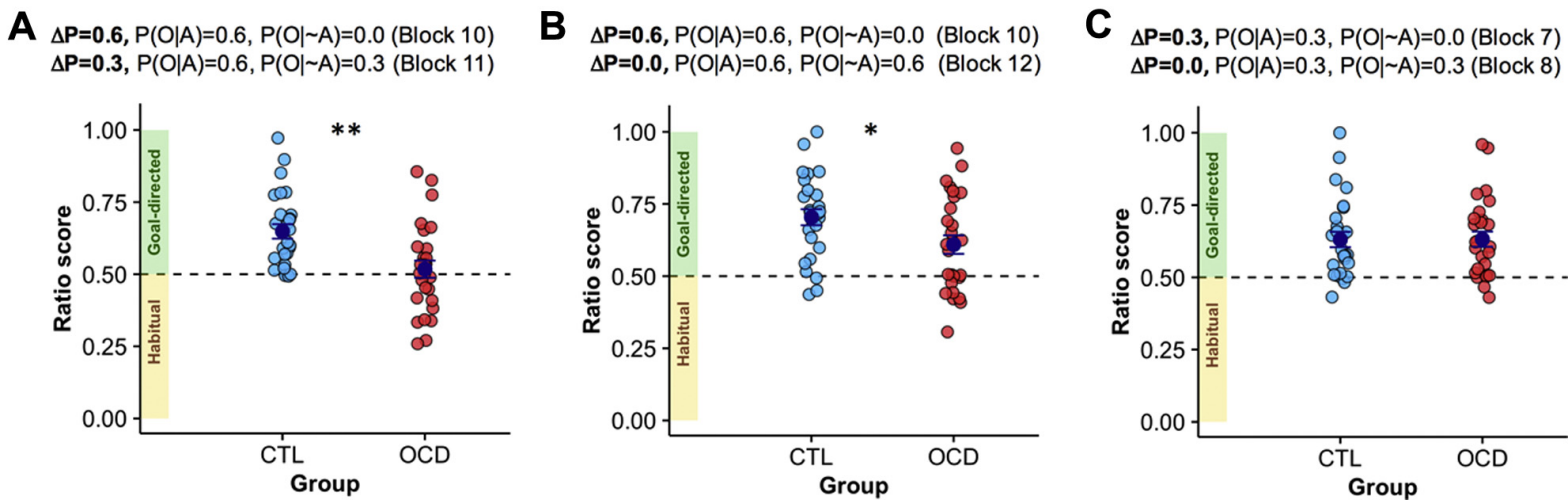
Habit and goal-directed ratio scores for contingent and corresponding degraded-contingency conditions. The ratio score was computed by dividing the number of responses in the nondegraded block by the sum of the responses for the degraded and nondegraded sessions [i.e., (contingent/(contingent+degraded)]. The ratio score represents the proportion of responses in the contingent condition relative to the nondegraded condition, with a value ≤0.5 indicating a greater or equal number of responses in the degraded contingency (ΔP) condition and thus habitual behavior. **(A)** Ratio score for pairs of blocks for which the action-outcome relationship was contingent (ΔP = 0.6 [P(O|A) = 0.6, P(O|∼A) = 0.0], block 10) and then degraded to ΔP = 0.3 by superimposing a noncontingent schedule (ΔP = 0.3 [P(O|A) = 0.6, P(O|∼A) = 0.3], block 11). Patients with obsessive-compulsive disorder (OCD) displayed increased habitual behavior (*t*_52_ = 3.350, *p* = .002). **(B)** Ratio score for pairs of blocks for which the action-outcome relationship was contingent (ΔP = 0.6 [P(O|A) = 0.6, P(O|∼A) = 0.0], block 10) and then completely degraded to ΔP = 0.0 by superimposing a noncontingent schedule (ΔP = 0.0 [P(O|A) = 0.6, P(O|∼A) = 0.6], block 12). Patients with OCD showed increased habitual behavior compared with control (CTL) subjects (*t*_52_ = 2.23, *p* = .03). **(C)** Ratio score for pairs of blocks for which the action-outcome relationship was contingent (ΔP = 0.3 [P(O|A) = 0.3, P(O|∼A) = 0.0], block 7) and then completely degraded to ΔP = 0.0 by superimposing a noncontingent schedule (ΔP = 0.0 [P(O|A) = 0.3, P(O|∼A) = 0.3], block 8). Error bars: SEM. *p < .05; **p < .01. P(O|A), probability of outcome given an action; P(O|∼A), probability of outcome given the absence of an action.

We computed a ratio score controlling for response variability across subjects and to test precisely if increased responding observed for ΔP = 0.3 (Figure 3A) was due to habitual behavior. We focused on contingency degradation occurring after the implicit training phase and computed the ratio score for pairs of blocks for which the action-outcome relationship was contingent (ΔP = 0.6 [P(O|A) = 0.6, P(O|∼A) = 0.0], block 10) and then degraded to ΔP = 0.3 by superimposing a noncontingent schedule (ΔP = 0.3 [P(O|A) = 0.6, P(O|∼A) = 0.3], block 11). Whereas control subjects showed a robust decline in responding on contingency degradation, as indicated by a ratio score well above 0.5 (one-sample *t* test tested against 0.5, *t*_26_ = 5.918, *p* < .001), patients responded nearly equally in both conditions, with their ratio score being close to 0.5 (one-sample *t* test against 0.5, *t*_26_ = 0.585, p = .563). There was a between-group difference in the ratio score (*t*_52_ = 3.350, *p* = .002) (Figure 4A). Furthermore, subjects were classified dichotomously as goal-directed (ratio score >0.5) or habitual (ratio score ≤0.5), with higher proportion of habitual subjects in the OCD group (control group, habitual 2/27; OCD group, habitual 12/27; χ^2^_1_ = 7.811, *p* = .005). There was no correlation between the ratio score and symptom severity (Yale-Brown Obsessive Compulsive Scale) in patients (*r* = −.101, *p* = .625).

Similarly, we observed a marginal effect for increased responding at ΔP = 0.0 (Figure 3A). Therefore, we calculated a ratio score for blocks for which the action outcome relationship was contingent (ΔP = 0.6 [P(O|A) = 0.6, P(O|∼A) = 0.0], block 10) and then completely degraded to ΔP = 0.0 by superimposing a noncontingent schedule (ΔP = 0.0 [P(O|A) = 0.6, P(O|∼A) = 0.6], block 12). Even though both control subjects and patients with OCD showed a response rate significantly different from 0.5 (one-sample *t* test against 0.5; control subjects: *t*_26_ = 7.334, *p* < .001; patients with OCD: *t*_26_ = 3.388, *p* = .002), patients showed diminished goal-directed behavior compared with control subjects (ratio score: *t*_52_ = 2.23, *p* = .03) (Figure 4B). There was no between-group difference when action-outcome relationship was completely degraded from low instrumental contingency (i.e., ΔP = 0.3 [P(O|A) = 0.3, P(O|∼A) = 0.0], block 7, and ΔP = 0.0 [P(O|A) = 0.3, P(O|∼A) = 0.3], block 8) (Figure 4C). The absence of this effect might be due to impaired detection of diminished instrumental contingency in patients. Patients were already showing increased responding for low instrumental contingencies compared with control subjects (ΔP = 0.3 [P(O|A) = 0.3, P(O|∼A) = 0.0], block 7: *F*_1,52_ = 4.961, *p* = .030) for which contingency degradation was therefore perhaps ineffective owing to a ceiling effect.

We did not detect an effect of repetition on habit development (Supplement) (36). Across groups, habitual behavior early in training was associated with higher OCD traits measured by the Obsessive-Compulsive Inventory–Revised (*r* = −.280, *p* = .046).

### Absence of Depressive Realism in OCD

**Figure 5:**
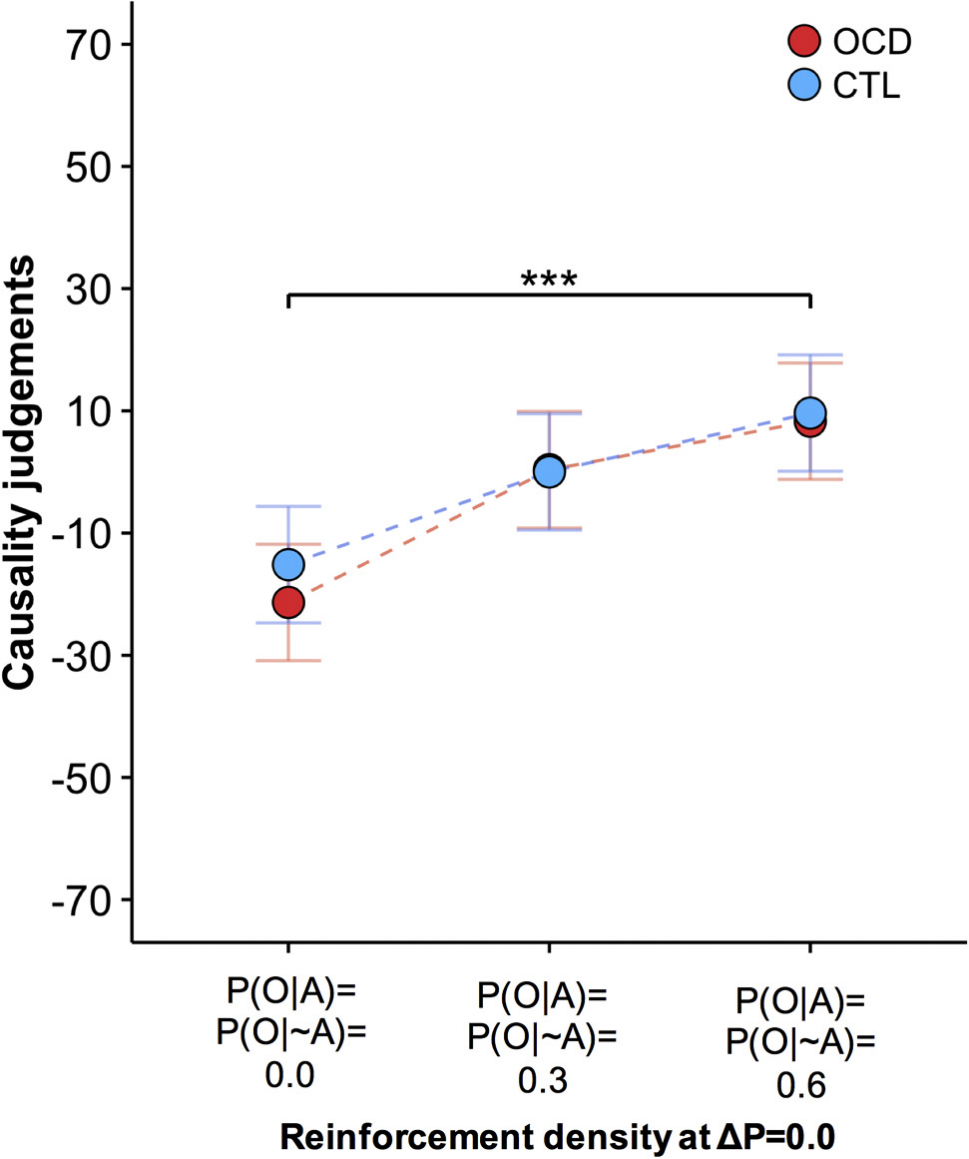
Causality judgments when the contingency (ΔP) was zero. There were no group differences. For both the control (CTL) group and obsessive-compulsive disorder (OCD) group, causality judgments increased as a function of higher density of reinforcement even though there was no causal association between the action and the outcome (contingency ΔP = 0.0) in all three situations. Error bar indicates Fisher’s least significant difference to facilitate post hoc comparisons (error bars are ± 0.5 least significant difference). However, in the context of mixed designs, as in this case, this error bar can be used only for within-subject comparisons. ***p ≤ .001, main effect of density of outcome. P(O|A), probability of outcome given an action; P(O|∼A), probability of outcome given the absence of an action.

Previous data have shown that healthy subjects have biased higher causality judgment estimates when the contingency is zero (37). This erroneous estimation arises when contingent and noncontingent outcomes occur frequently (i.e., high density of reinforcement), but not when they occur infrequently (i.e., low density of reinforcement). In contrast, depressed individuals show a depressive realism whereby, regardless of the density of reinforcement, they correctly report having no causal effect on outcome occurrence (37). Because patients showed higher depression scores compared with control subjects, we tested between-group differences in causality judgments for ΔP = 0.0 blocks with different reinforcement densities (blocks 4, 8, and 12) (Table 2). Selection was limited to the Latin square phase to have an equal number of observations for each condition. Estimation of causal control was higher for higher reinforcement density (*F*_2,104_ = 8.365, *p* < .001) (Figure 5A), with no between-group differences (group: *F*_1,52_ = 0.171, *p* = .681; group × reward density; *F*_2,104_ = 0.124, *p* = .883), despite higher depressive symptoms in patients.

## Discussion

Patients with OCD showed dissociations between their exhibited response rates and their causal judgments about the effectiveness in controlling outcomes. This was manifested in distinct ways for positive and negative contingencies. For positive contingencies, OCD patients exhibited increased response rates when outcomes were less contingent on responding. This lack of influence of instrumental contingency over behavior is consistent with the overresponding resulting from enhanced S-R habitual tendencies and diminished sensitivity to instrumental contingency. In contrast, explicit action-outcome knowledge was intact: patients gave accurate subjective assessments of the cause-effect relationship between actions and their consequences, which did not differ from those of control subjects. However, patients used their knowledge about the environmental contingencies to guide their actions in a manner that differed from control subjects. Hence, in patients with OCD, for positive contingencies, excessive responding was dissociated from intact explicit action-outcome knowledge. For negative contingencies, the interaction was reversed between response rate and subjective judgment. Here, response rates were equivalent between the two groups, despite patients with OCD judging their actions to be somewhat less detrimental.

In previous neuroimaging studies of contingency knowledge in healthy volunteers, functional activity of the inferior and superior parietal lobule and the middle frontal gyrus was shown to scale with subjective reports of instrumental contingency (19). In OCD, parietal abnormalities (38) together with diminished caudate-parietal connectivity 39, 40 might contribute to the inefficient use of explicit knowledge of instrumental contingencies to guide behavior. The inability to modulate behavior according to action-outcome contingencies in patients might be due not only to abnormal striatal encoding of action-outcome contingencies but also (or alternatively) to an inability of action-outcome metacognitive knowledge (putatively dependent on parietal activity) to guide responding. In this respect, future empirical testing using functional magnetic resonance imaging would clarify whether a lack of integration between the frontoparietal system and the caudate contributes to the ego-dystonic, compulsive nature of OCD.

Accurate subjective judgments, especially for positive contingencies, indicated intact action-outcome knowledge not only in control subjects, as previously shown 14, 19, 20, 21, but also in patients with OCD. For negative contingencies only, there were subjective judgment inaccuracies in patients, as actions were reported as less detrimental than experienced. Although these findings with negative contingencies should be interpreted with caution in the context of a lack of a main group effect, they suggest that patients with OCD might perceive their actions to have less disadvantageous consequences than experienced. Overall, when noncontingent outcomes were more likely than contingent ones, increased response rates and inaccurate contingency ratings were observed. Even though it remains to be clarified why noncontingent outcomes had a differential effect on behavior and causality judgments, patients had particular difficulties in integrating noncontingent conditional probabilities. Such an effect might depend on a circuit including the posterior caudate and the inferior frontal gyrus, which has been shown to selectively decode noncontingent conditional probabilities (19).

More generally, cognitive theories of OCD 7, 41 point to an exaggerated appraisal of intrusive thoughts, which is believed to be critical in the maintenance of the disorder. In this respect, OCD is conceptualized in terms of the impact of inflated evaluation of intrusive thoughts on action. In direct contrast, in the present study patients with OCD showed intact action-outcome contingency knowledge, especially for positive contingencies; however, despite this correct contingency appraisal, they exhibited exaggerated responding. Therefore, rather than supporting a model whereby OCD is maintained by exaggerated and dysfunctional appraisal of action contingencies, the findings suggest that exaggerated actions, possibly rooted in a propensity toward habits (42), lie at the core of the disorder.

Patients exhibited excessive responding when the action-outcome contingency was degraded, the effect being present when contingency was partially and completely degraded. Excessive responding emerged both from insensitivity to free rewards, presumably owing to habitual responding (Figure 4), and from decreased sensitivity to low instrumental contingencies (Figure 3), presumably deriving from a deficient goal-directed system. Our findings resonate with previous studies that used outcome devaluation in appetitive (9) and aversive domains (11). We extended those findings by testing habits via contingency degradation (1). Correct action-outcome contingency appraisal agrees with previous data showing intact awareness of explicit associative contingencies in the case of outcome devaluation in OCD (11), although in a context of multiple action-outcome associations, patients with OCD show weaker knowledge of action-outcome causal relationships (9). Neurocomputational models have also suggested imbalances between the goal-directed and the habitual systems in OCD (25).

Translational work using contingency degradation in rats 13, 16, 17, marmoset monkeys (18), and humans 14, 19, 21 has identified corticostriatal determinants of goal-directed and habitual actions. In rats, lesions of the prelimbic cortex and the dorsomedial striatum (the putative homologue of the human caudate nucleus) prevented action-outcome association encoding during instrumental conditioning (43). In marmosets, insensitivity to contingency degradation was detected following perigenual anterior cingulate and orbitofrontal cortex lesions (18). In humans, activity in the medial prefrontal cortex/medial orbital cortex and the anterior caudate was associated with contingency learning and goal-directed behavior 19, 21, 44. In healthy volunteers, reduced gray matter volume in the caudate correlated with a propensity toward habits (25). In OCD, a hyperactive caudate nucleus was also related to excessive habit formation, tested in avoidance with outcome devaluation (10). Therefore, lack of behavioral suppression on contingency degradation plausibly depends on abnormalities in circuits also implicated in OCD pathophysiology (23). Animal work has also shown differential sensitivity to outcome devaluation and contingency degradation (45). Such a distinction can be now tested in humans by using the paradigm devised here as well as those using outcome devaluation.

Previous studies have shown that affective states influence the way contingencies are perceived (37). When there is a lack of action-outcome contingency and noncontingent reward occurs frequently, causal control overestimation is observed in nondepressed people. Depressed individuals show instead an accurate detection of the lack of contingency (i.e., depressive realism). In this study, when the contingency was zero, causality judgments increased as a function of the reward density, equally in control subjects and patients. Even though patients were relatively more depressed than control subjects, their emotional/affective state did not influence their contingencies’ perception in a way that was significantly different from that of control subjects.

Classical theories predict habit development owing to repetition over time (36), but we did not observe a shift from goal-directed to habitual behavior over early and late experimental phases. This might be due to the experimental design, which did not lend itself to optimal investigation of this possibility. At the beginning, participants experienced the first three blocks in the same order, but then blocks were presented in a semirandomized design. This manipulation might conceivably have diluted the effect of repetition owing to the different number of instrumental contingencies experienced before the relevant critical test across subjects. Furthermore, the short task duration may have limited the possibility of detecting training effects, in line with evidence that limited overtraining in instrumental behaviors often fails to produce habit learning (46).

OCD is known to be linked to serotonergic abnormalities, and there is evidence in healthy humans that diminished serotonin promotes habitual behavior (47). The effect we observed was not apparently due to patients’ medication status; such medication is designed to increase serotonergic transmission. This conclusion, however, has limited statistical power owing to the small sample size.

In conclusion, this study shows a mismatch between explicit action-outcome knowledge and behavior, possibly reflecting the ego-dystonic nature of OCD. It reinforces the hypothesis that a deficient goal-directed system is a contributor to OCD, using the criterion of contingency degradation and employing a novel, translationally valid behavioral paradigm.
