# Supplementary material for: Action-Outcome Knowledge Dissociates From Behavior in Obsessive-Compulsive Disorder Following Contingency Degradation
Source: Biol Psychiatry Cogn Neurosci Neuroimaging. 2019 Feb;4(2):200–9. doi: 10.1016/j.bpsc.2018.09.014 (PMC6374986; doi:10.1016/j.bpsc.2018.09.014)
Supplement: Supplemental Material [file mmc1.pdf]

Action-Outcome Knowledge Dissociates From Behavior in Obsessive-Compulsive Disorder Following Contingency Degradation

Supplementary Information

Supplementary Methods & Materials

Participants

Control subjects were recruited from the community; none of them was on psychiatric medication and they never suffered from a psychiatric disorder. Patients were recruited through clinical referral from local psychiatric and psychological services or local advertisement. In addition, patients who participated in previous independent studies were contacted by phone. A fully certified consultant psychiatrist made DSM-5 diagnoses using an extended clinical interview, supplemented by the Mini International Neuropsychiatric Interview (1). Self-reported measures of anxiety were collected using the State-Trait Anxiety Inventory (STAI) (2); and, in addition to Y-BOCS scores, self-reported measures of OCD symptomatology were collected using the Obsessive Compulsive Inventory-Revised (OCI-R) (3). Exclusion criteria for all participants were: current substance dependence, head injury, and current depression, indexed by Montgomery-Åsberg Depression Rating Scale exceeding 16 (4) during screening. Depression and anxiety symptoms were below threshold for depressive or anxiety disorder diagnosis. 19 of the 27 patients were taking stable doses of serotonin reuptake inhibitors (SSRIs) medication for a minimum of 8 weeks prior taking part in the study. As an adjunct to their SSRIs, 3 of these patients were taking antipsychotics (Quetiapine). The remaining 8 patients were unmedicated, being either drug- naïve or off medication for at least 8 weeks prior taking part of the study. Most of the participants completed two other behavioural tasks, unrelated to the present study. The study was approved by the NHS East of England, Cambridge Central Research Ethics Committee. Participants were reimbursed for their time and informed consent was obtained prior participation.

No statistical methods were used to pre-determine sample size but our sample sizes are similar to those generally employed in the field, with power of 0.8 to detect effect sizes of 0.78 at α=0.05, two-tailed.

A novel protocol to test sensitivity to action-outcome contingency

The instructions informed the participants that they could earn 25 pence (p; £0.25) whilst pressing the space bar on a keyboard, and that they were free to press the key as often as they liked (Figure 2A and Methods and Materials). They were further instructed that the relationship between pressing the space bar and receiving the 25p reward would vary during the experiment, and that pressing the space bar might earn a reward, a reward might also arrive on its own, or pressing the space bar might prevent a reward from arriving. Lastly, they were informed that occasionally they would be asked to rate the degree to which pressing the space bar caused the occurrence of the reward. By varying P(O|A) and P(O|~A) we obtained blocks with different levels of contingency and thus different experimental conditions (Figure 2B, C and Table 2). In positive contingency conditions, P(O|A) was higher than P(O|~A). Those were degraded by increasing P(O|~A). To mimic the maladaptive nature of compulsivity in OCD, by which actions are repeated despite adverse consequences, negative contingencies were also introduced in the experimental paradigm whereby P(O|~A) was higher than P(O|A). In these situations, performing the action reduced the probability of getting an outcome. Our implementation of the task differed from previous ones available in the literature for some crucial aspects. Firstly, we used unsignalled time bins and we specified the conditional probabilities a priori. This ensured that experienced instrumental contingencies did not deviate substantially from the programmed ones. In agreement with previous implementations, we adopted a free-operant, self-paced procedure whereby the participant could decide whether to press the space bar or not when presented with a white triangle on the screen. However, in a free-operant paradigm, the degree of contingency experienced can be determined partly by the behavior, and experienced

contingency might in principle vary substantially across participants (e.g., someone who never responds would never experience P(O|A), and someone who never ceases responding would never experience P(O|~A)). In schedules where reinforcer delivery is influenced by time (e.g. with a maximum reinforcer delivery rate or on an interval schedule), different subjects might experience similar reinforcer delivery rates despite different response rates. Therefore, we divided time into short 1 second interval (bin), and calculated 'response' versus 'no response' on a per- bin basis ensuring a close correspondence between programmed and experienced contingencies (5). Accordingly, unbeknown to the participant each block was divided into bins, treated as a trial by the experimenter. The procedure was free-operant for the subject as trials were unsignaled and there was no inter-trial interval. In doing so, interpretation of our findings was not confounded by between-groups differences in experienced contingencies (Table 2).

Secondly, in line with experimental studies in rodents where there is no explicit 'punishment' for responding we did not include a cost for responding (see Supplementary Pilot experiments and Figure S3 for supporting results from pilot experiments with and without such costs). We found that introducing a cost induced a generalized reduction of responding, with no specific effect on determining responding in face of degradation and therefore opted for a version most similar to translational implementations. The experiment was programmed using Psychtoolbox 3 (6).The overall duration of the task was variable due to its free-operant nature, i.e. the rate of responding which was variable across participants determined the number of outcomes. In fact, we had a fixed amount of unsignalled bins for each block but delivery of a reinforcer delayed the start of the next bin. Hence the total duration depended also on the number of outcomes delivered but the average time for completion (34 minutes) did not differ between groups.

Supplementary Results

Experienced contingency

In order to compute the mean experienced contingency for each subject for a given block, we recorded (i) the number of contingent outcomes (rewards delivered upon key press) (C1); (ii) the number of times that a key press was not associated with the delivery of an outcome (C2); (iii) the number of non-contingent outcomes (rewards delivered in the absence of a key press) (C3); (iv) the number of times that there was no key press and no outcome delivered (C4). We thus computed the experienced contingency based on the formula for contingency (DP) (4):

$$\Delta P = (O|A) - P(O|{\sim}A)$$

as:

$$\left[\frac{\mathrm{C1}}{(\mathrm{C1}+\mathrm{C2})}\right] - \left[\frac{\mathrm{C3}}{(\mathrm{C3}+\mathrm{C4})}\right]$$

In very few instances experienced contingency could not be computed because there were no occurrences of either C1 and C2 or C3 and C4. In other words, the subject did not press the space bar throughout the block, or adopted a constant pressing rate with a consequential lack of no trials with no responses. However, in our entire data set (648 blocks; 12 blocks x 54 participants) this occurred only on 10 single occasions with 7 controls and 3 OCD patients adopting one of the specified strategies in one of the blocks during their experimental session. Inclusion or exclusion of these subjects did not affected the main findings, therefore, we retained data from these subjects for the analysis.

Effect of medication on response rate and causality judgements

For response rate, the group difference in the effect of contingency remained significant even when considering only medicated OCD and controls ($\text{group}_{\text{OCD medicated, Controls}}$ ×contingency, $F_{4,176}$=4.107, p=0.003) or only unmedicated OCD and controls (group $_{\text{OCD}}$

$_{\text{unmedicated, Controls}}$ ×contingency, $F_{4,132}$=2.628, p=0.037). There were no between-group effects nor interactions that depended on medication status in OCD patients (all p > 0.1) (**Figure S1**). Similarly, for causality judgments, the results did not change when considering only medicated or unmedicated OCD versus controls. There were no between-group effects nor interactions that depended on medication status in patients (all p >0.186) (**Figure S1**).

As expected, there was a main effect of programmed contingency on the number of outcomes obtained ($F_{4,208}$=38.831, p<0.001). Even though OCD patients responded more at certain levels of instrumental contingencies, such increased behavior was not sufficient to lead to a higher number of obtained outcomes. In fact, there was no main effect of group on the number of outcomes obtained ($F_{1,52}$=0.002, p=0.960), nor a significant interaction between group and programmed contingency ($F_{4,208}$=1.158, p=0.330). These findings therefore rule out the possibilities that OCD patients' behavior resulted in better outcomes overall or that OCD patients' behavior was secondary to differences in reward rate. In addition, we used the BIS/BAS (Behavioral Inhibition System/Behavioral Approach System) questionnaire to measure reward responsiveness via the BAS reward responsiveness subscale (7). Although data were available only for a subset of subjects (18 controls and 19 OCD) there was no group difference in reward responsiveness (t_{35}=0.375, p=0.710). There was no difference in response rate at the maximal contingency (**Figure 3A**, at ∆P=0.6), but specifically for certain levels of contingency suggesting that the effect was due to reasons other than reward responsiveness.

Relationship between response rate and causality judgments

We excluded that OCD patients were simply slower to learn the new contingency by analyzing response rate for different time windows. In other words, we divided each block in three parts comprising trials 1-40 (first time window), 41-80 (second time window), 81-120 (third time window). In the first time window, mean response rate increased with

contingency (contingency, $F_{4,208}$=22.17, p<0.001). The two groups did not differ in their response rate (group, $F_{1,52}$=0.759, p=0.388; group×contingency, $F_{4,208}$=0.899, p=0.465) (**Figure S2A**). In the second window, mean response rate increased with contingency (contingency, $F_{4,208}$=54.724, p<0.001). Overall levels of responding did not differ between the groups (effect of group on response rate, $F_{1,52}$=0.874, p=0.354). Responding in the groups was differentially affected by the contingency (group×contingency, $F_{4,208}$=3.674, p=0.006); this difference was explored via between-groups simple-effect comparisons at each level of contingency. Patients with OCD persisted in responding more than healthy subjects in face of low instrumental contingency (group effect at DP=0.3; $F_{1,52}$=5.645, p=0.021). Patients responded marginally more at DP =0.0, but this did not reach significance ($F_{1,52}$=3.636, p=0.062) (**Figure S2B**). In the third time window, mean response rate increased with contingency (contingency, $F_{4,208}$=66.289, p<0.001) (**Figure S2C**). Overall levels of responding did not differ between the groups (effect of group on response rate, $F_{1,52}$=1.534, p=0.221). Responding in the groups was differentially affected by the contingency (group×contingency, $F_{4,208}$=4.736, p=0.001); this difference was explored via between-groups simple-effect comparisons at each level of contingency. Patients persisted in responding more than healthy subjects in face of low instrumental contingency (group effect at DP=0.3; $F_{1,52}$=8.340, p=0.006). Even at DP =0.0 OCD persisted in responding more than healthy subjects in face of low instrumental contingency (group effect at DP =0.0; $F_{1,52}$=4.410, p=0.041). Therefore, habitual responding emerged especially in later time windows, closer to causality judgments.

This analysis rules out that OCD patients were simply slower to learn the contingency, and supports the claim of a dissociation whereby habitual responding was observed in temporal time windows closer to causality judgments rating, for which OCD patients did not differ from controls.

Habit/goal-directed ration score, effect of repetition

To test the effect of repetition in the development of habits, we computed the ratio score for the early phases of the experimental design (Early: Block 1 and Block 2) and compared with late ones (Late: Block 10 and Block 12). There was no main effect of time ($F_{1,52}$=0.083, p=0.775) nor a time × group interaction ($F_{1,52}$=0.648, p=0.425).

Supplementary pilot experiments

We piloted two different versions of the experiment to particularly assess whether a cost for responding should be included. We performed two pilot experiments which included the same experimental conditions, but differed for the presence of a cost associated with pressing. For both versions, we programmed conditional probabilities as follows (Block 1: DP=0.2, P(O|A)=0.2, P(O|~A)=0.0; Block 2: DP=0.0, P(O|A)=0.2, P(O|~A)=0.2; Block 3: DP=0.2, P(O|A)=0.2, P(O|~A)=0.0; Block 4: DP=0.1, P(O|A)=0.1, P(O|~A)=0.0; Block 5: DP=0.0, P(O|A)=0.1, P(O|~A)=0.1; Block 6: DP=0.1, P(O|A)=0.1, P(O|~A)=0.0). The only difference between the two experiments is that in one case we included a cost of £ 0.01 for responding, while in the other case there was no cost associated with responding. We had 5 subjects for the version with a cost associated with responding and 11 subjects for the version with no cost associated with responding. All the subjects were healthy volunteers recruited from the community, none of them was on psychiatric medication and they never suffered from a psychiatric disorder. The task had the same structure as the one presented in the main manuscript the only difference consisting in the underlying densities of the conditional probabilities and the number of blocks. We analyzed performance in terms of response for different blocks. To measure behavioral sensitivity to instrumental contingency we computed a response rate, obtained by dividing the number of responses by the number of bins for each block. For response rate, block was used as a within-subject factor and the version of the task as a between-subject factor. Analyses were performed in R version 3.3.1

(http://www.r-project.org/) using the ‘ez’ package for ANOVA. Levene’s test was used to verify homogeneity of variance. Mauchly’s test of sphericity was applied and Greenhouse–Geisser and Huynh–Feldt correction used for substantial (e<0.75) and minimal violation (e<0.75), respectively. There was a main effect of version on response rate ($F_{1,14}$=10.781, p=0.005) and a main effect of block ($F_{5,70}$=12.422, p<0.001) but not a significant Version by Block interaction (**Figure S3**). The main effect of version on response rate showed that responses were generally dampened in the case of a cost for responding, however the absence of an interaction with block showed that this effect was generalized and not specific to degraded or non-degraded blocks. The main effect of block shows that subjects were able to modulate responding according to whether the contingency was degraded or not with lower response rates for degraded conditions, regardless of the presence of a cost associated with responding. Therefore, these findings led us to the conclusion that an explicit cost for responding is associated with reduced responding overall but it does not specifically impact on the evaluation of responding in case of contingency degradation. Because there was a reduction in response rate in the degraded condition with or without response costs, response cost is not essential for modulating response rate in the degraded conditions. There may also be implicit costs to responding (e.g. effort), in light of these findings we did not include explicit response costs in the main task, to reduce the additional cognitive burden of explicit cost/benefit analysis during performance. Additionally, the reduction in overall responding caused by explicit response costs has the potential to lead to under-exploration of the instrumental contingencies.

Supplementary Figures

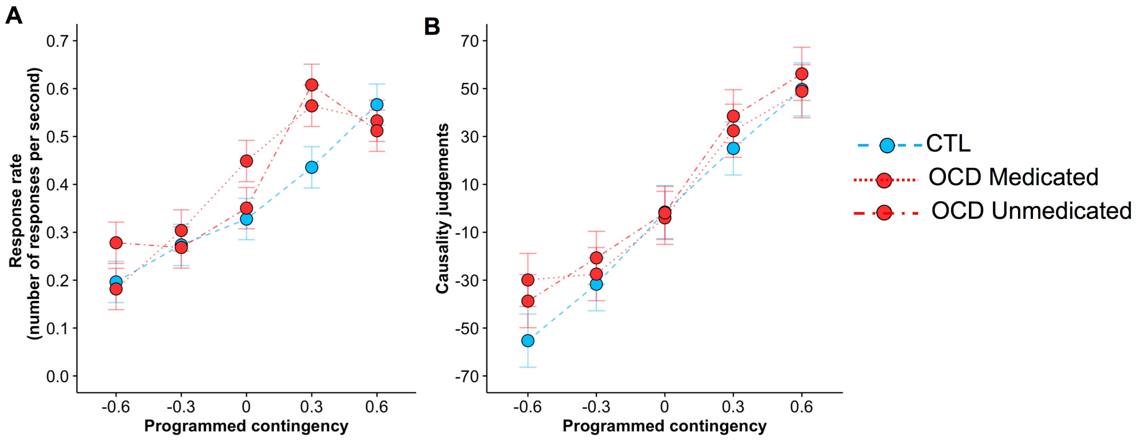

Figure S1. Increased response rate but intact action-outcome knowledge in OCD patients regardless of medication. (A) Mean response rate by contingency. Groups responded more for higher contingencies. However, OCD patients, regardless of medication, showed reduced sensitivity to instrumental contingency. See main text for statistics. **(B)** Subjective judgments of causality increased as a direct function of response–outcome contingency in the three groups without any significant difference between medicated and unmedicated OCD patients. Data are presented in ascending order of programmed contingency, but contingencies were experienced by each subject in a semi-randomized order. Error bar indicates Fisher's Least Significant Difference (FLSD) to facilitate post-hoc comparisons (error bars are $\pm$ 0.5 x $t_{critical}$ x SD). However, in the context of mixed designs, as in this case, this error bar can only be used for within-subject comparisons. CTL, controls; OCD, patients with obsessive–compulsive disorder. Please note that as described in the main text data were collapsed across blocks having equal contingencies [ΔP = -0.6, Block 6; ΔP= -0.3, Block 5, Block 9; ΔP=0.0, Block 2, Block 3, Block 4, Block 8, Block 12; ΔP=0.3, Block 7, Block 11; ΔP=0.6, Block 1, Block 10. See Table 1 for naming of the blocks]. Programmed contingency refers to the a priori experimentally programmed contingency.

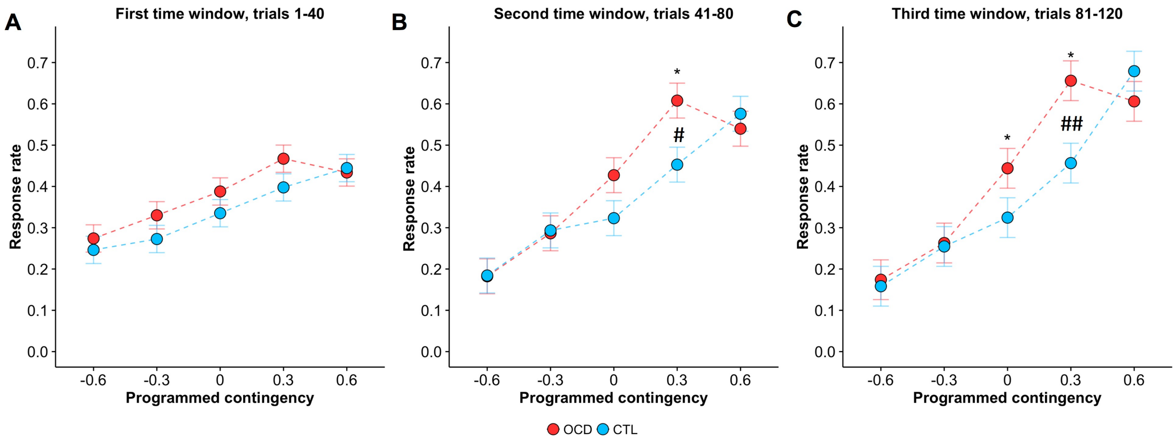

Figure S2. Mean response rate for controls and OCD patients for different time windows of the block. (A) Mean response rate by contingency for the first time window of each block (comprising trials 1-40). Both groups responded more for higher contingencies. With no differences between groups. **(B)** Mean response rate by contingency for the second time window of each block (comprising trials 41-80). Both groups responded more for higher contingencies. However, OCD patients showed reduced sensitivity to instrumental contingency. #p£0.01, interaction; *p£0.05, for between-group comparison. **(C)** Mean response rate by contingency for the second time window of each block (comprising trials 81-120). Both groups responded more for higher contingencies. However, OCD patients showed reduced sensitivity to instrumental contingency. ##p£0.01, interaction; *p£0.05, for between-group comparison. Error bar indicates Fisher's Least Significant Difference (FLSD) to facilitate post-hoc comparisons (error bars are ± 0.5 x $t_{critical}$ x SD). However, in the context of mixed designs, as in this case, this error bar can only be used for within-subject comparisons. CTL, controls; OCD, patients with obsessive–compulsive disorder. Please note that as described in the main text data were collapsed across blocks having equal contingencies [∆P = -0.6, Block 6; ∆P = -0.3, Block 5, Block 9; ∆P = 0.0, Block 2, Block 3 Block 4, Block 8, Block 12; ∆P = 0.3, Block 7, Block 11; ∆P = 0.6, Block 1, Block 10. See Table 2 for naming of the blocks].

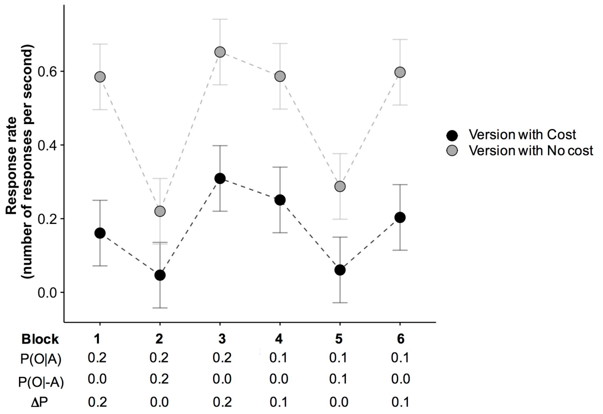

Figure S3. Response rate for pilot versions of the experiment with and without response costs. Mean response rate by block for separate versions of the experiment including a cost for responding (black, Version with cost) and for version of the experiment without a cost for responding (grey, Version with No cost). Each version included six blocks (see Supplementary pilot experiments). Responses costs affected responding, as did block (contingency), but response costs did not affect the impact of contingency manipulations (see Supplementary pilot experiments).

Supplementary References

1. Sheehan DV, Lecrubier Y, Sheehan KH, Amorim P, Janavs J, Weiller E, *et al.* (1998): The Mini-International Neuropsychiatric Interview (M.I.N.I.): the development and validation of a structured diagnostic psychiatric interview for DSM-IV and ICD-10. *J Clin Psychiatry*. 59 Suppl 20: 22-33;quiz 34-57.
2. Spielberger CD (1983): *Manual for the State-Trait Anxiety Inventory (STAI).* Palo Alto, CA: Consulting Psychologists Press.
3. Wootton BM, Diefenbach GJ, Bragdon LB, Steketee G, Frost RO, Tolin DF (2015): A contemporary psychometric evaluation of the obsessive compulsive inventory-revised (OCI-r). *Psychol Assess*. . doi: 10.1037/pas0000075.
4. Montgomery SA, Asberg M (1979): A new depression scale designed to be sensitive to change. *Br J Psychiatry J Ment Sci*. 134: 382–389.
5. Hammond LJ (1980): The effect of contingency upon the appetitive conditioning of free-operant behavior. *J Exp Anal Behav*. 34: 297–304.
6. Kleiner M, Brainard D, Pelli D, Ingling A, Murray R, Broussard C (2007): What's new in psychtoolbox-3. *Perception*. 36: 1–16.
7. Carver, C. S., & White, T. L. (1994): Behavioral inhibition, behavioral activation, and affective responses to impending reward and punishment: The BIS/BAS Scales. *Journal of Personality and Social Psychology,*. 67: 319–333.
